# Postnatal depression and bonding disorder: A tautology or two overlaping phenomena?

**DOI:** 10.1192/j.eurpsy.2025.811

**Published:** 2025-08-26

**Authors:** G. Hernández-Santillán, M. Gurpegui, M. Alcamí-Pertejo, G. Lahera, M. F. Bravo-Ortiz

**Affiliations:** 1 Psychiatry, Hospital Universitario La Paz, Madrid; 2 Granada Centre for Psychiatric Studies, Granada; 3 Psychiatry, Hospital Universitario Príncipe de Asturias; 4 Psychiatry, Universidad de Alcalá, Madrid, Spain

## Abstract

**Introduction:**

Perinatal disorders occur in 25% of childbearing women. Postnatal depressive symptoms (PDS) have been widely studied, whilst PDS usually overshadows bonding disorder (BD) in clinical practice and research. BD includes mild disorders, such as delay, ambivalence or loss of maternal emotional response, and severe disorders, such as pathological anger or rejection of the child (Brockington et al., Arch Womens Ment Health 2006; 9(5) 243-251).

**Objectives:**

To estimate the prevalences of PDS and BD in mothers during the six months after birth.

**Methods:**

Women and their male partners aged ≥ 18, without delivery and neonatal complications, were recruited at the Maternity Ward in a public hospital in Madrid, during 2021-2022. Data was collected at immediate puerperium (T0), sixth week (T1), fourth month (T2), and sixth month (T3). The last observation carried forward (LOCF) was used. An Ad hoc Socio-Demographic questionnaire was used. To determine the presence of PDS and BD, respectively, there were used the Edinburgh Postnatal Depression Scale (EPDS), cut off ≥ 11 (Ascaso-Terrén et al., Med Clin (Barc) 2003; 120(9) 326-329) and Postpartum Bonding Questionnaire (PBQ), cut off ≥ 13 for BD, and ≥ 18 for severe BD (Torres-Giménez et al., Span J Psychol. 2021; 24, e47, 1-9).

**Results:**

1502 couples were recruited at T0. The main characteristics of female participants were: mean age 34.1 years, 53.9% married, 54.1% primiparous, 27.8% migrants, 67.3% university degree or higher, 83.2% employed, 14.8% financial difficulties, 4.9% smoking during pregnancy and, 21.7% c-section. At T0, the prevalences of PDS were 13.0% of mothers, 10.5% of fathers, and 3.5% of both parents. Applying LOCF, 874 women responded to the questionnaires at some timing during the follow-up. The results were divided into two groups (see Table 1 and Table 2) depending on whether they presented PDS at T0. In mothers with PDS at T0, PDS and BD rates eventually decrease at T3. In the other group, while BD rates decrease at T3, a slight increase in PDS presentation at T3 is observed.

**Table 1.** LOCF of mothers with PDS at T0

N	Follow-up	No-PDS	BD	Severe BD
106	T1	53/87 (60.9%)	49/87 (53.3%)	32/87 (36.78%)
PDS at T0	T2	47/74 (63.5%)	33/74 (44.6%)	19/74 (25.7%)
	T3	44/73 (60.3%)	30/73 (41.1%)	14/73 (19.2%)

**Table 2.** LOCF of mothers without PDS at T0

N	Follow-up	PDS	BD	Severe BD
768	T1	46/638 (7.2%)	225/638 (35.3%)	106/638 (16.6%)
No-PDS at T0	T2	47/575 (8.2%)	147/575 (25.6%)	66/575 (11.5%)
	T3	45/525 (8.6%)	122/525 (23.2%)	57/525 (10.9%)

**Conclusions:**

Depressive symptoms and impaired bonding could have different severity and timing during the postnatal period. More research on bonding disorder is needed to clarify more accurately the psychopathological features that distinguish it from postnatal depression to provide more targeted treatment that will also reduce the associated stigma of childbearing difficulties.

**Disclosure of Interest:**

None Declared

